# Optimized Ultrasound-Assisted Extraction Reveals *Salvia transsylvanica* as a Rosmarinic Acid-Rich Endemic Sage with Antioxidant Potential

**DOI:** 10.3390/antiox15050561

**Published:** 2026-04-28

**Authors:** Maria-Doroteia Brudiu, Alexandru Nicolescu, Adrian Gabriel Moroșan, Adriana Cristina Urcan, Laurian Vlase, Ana-Maria Vlase, Andrei Mocan, Gianina Crișan

**Affiliations:** 1 Department of Pharmaceutical Botany, Faculty of Pharmacy, “Iuliu Hațieganu” University of Medicine and Pharmacy, Gheorghe Marinescu Street 23, 400337 Cluj-Napoca, Romania; maria.doroteia.aloman@elearn.umfcluj.ro (M.-D.B.); morosan.adrian.gabriel@elearn.umfcluj.ro (A.G.M.); gheldiu.ana@umfcluj.ro (A.-M.V.); mocan.andrei@umfcluj.ro (A.M.); gcrisan@umfcluj.ro (G.C.); 2 Laboratory of Chromatography, Institute of Advanced Horticulture Research of Transylvania, Faculty of Horticulture and Business in Rural Development, University of Agricultural Sciences and Veterinary Medicine, Calea Mănăştur 3-5, 400372 Cluj-Napoca, Romania; 3 National Institute for Research and Development of Isotopic and Molecular Technologies, 67-103 Donat Street, 400293 Cluj-Napoca, Romania; 4 Department of Microbiology and Immunology, Faculty of Animal Science and Biotechnology, University of Agricultural Sciences and Veterinary Medicine Cluj-Napoca, 400372 Cluj-Napoca, Romania; adriana.urcan@usamvcluj.ro; 5 Department of Pharmaceutical Technology and Biopharmacy, Faculty of Pharmacy, “Iuliu Hațieganu” University of Medicine and Pharmacy, 41 Victor Babes Street, 400012 Cluj-Napoca, Romania; laurian.vlase@umfcluj.ro

**Keywords:** *Salvia transsylvanica*, endemic flora, ultrasound-assisted extraction, extraction optimization, metabolite accumulation, phenolic compounds, rosmarinic acid, antioxidant activity, HPLC-MS

## Abstract

*Salvia transsylvanica* was investigated as a source of bioactive metabolites by optimizing hydroethanolic ultrasound-assisted extraction (UAE) and comparing it with classical preparations. A D-optimal quadratic design was applied to aerial parts to evaluate the effects of ultrasonic amplitude, extraction time, and ethanol concentration on total phenolic content (TPC) and antioxidant capacity (ABTS, DPPH), yielding models with good fit and predictive ability. The optimal conditions (24% amplitude, 12 min, 38% ethanol) were then applied to aerial parts, flowers, and leaves, affording extracts with high TPC values (up to 240 mg GAE/g extract) and antioxidant activities comparable to aqueous infusions and 70% hydroethanolic macerates, with FRAP and DPPH values above 400 mg TE/g dw. Targeted HPLC-MS analysis revealed a *Salvia*-typical phenolic profile dominated by rosmarinic acid (up to 20 mg/g extract), methyl rosmarinate, caffeic acid derivatives, salvianolic acid K, and flavone glycosides (luteolin-7-*O*-glucoside and apigenin-7-*O*-glucoside), with leaf extracts generally richest in phenolics and antioxidant capacity. Conversely, macerates showed superior recovery of phytosterols and tocopherols. The extracts displayed moderate, selective antibacterial effects, particularly against *Staphylococcus aureus*, with inhibition zones up to 4 mm for flower-based preparations. Overall, UAE emerged as an efficient, green strategy for a rapid recovery of phenolic antioxidants from *S. transsylvanica*, while classical maceration complements it for lipophilic constituents, supporting the valorization of this endemic sage.

## 1. Introduction

*Salvia* L. (Lamiaceae) is one of the largest and most ethnobotanically significant genera in the plant kingdom, comprising approximately 1000 species distributed across temperate and subtropical regions worldwide [1]. Their relevance has been documented since antiquity, with classical preparations, especially aqueous infusions and hydroethanolic macerates, being employed for the management of gastrointestinal, respiratory, dermatological, and genitourinary ailments across diverse cultural traditions [1,2,3,4]. Constituent phenolic fractions, characteristically dominated by rosmarinic acid and related caffeic acid oligomers (including salvianolic acids), alongside flavone glycosides, are major contributors to bioactive effects (i.e., antioxidant, antimicrobial, anti-inflammatory, and hypoglycemic) [5,6,7,8].

Within Romanian ethnobotanical traditions, the most representative sage species are *S. officinalis*, *S. glutinosa*, *S. nemorosa*, *S. pratensis*, *S. aethiopis*, *S. sclarea*, and *S. verticillata* representing the most studied species of the national flora [1,2,3,4]. Recent pharmacognostical and phytochemical investigations of these species have confirmed rosmarinic acid as the major phenolic constituent across extraction methods, while indicating significant variability between species and plant organs [4,9,10,11,12,13,14,15].

*S. transsylvanica* (Schur ex Griseb. & Schenk) Schur—the “Transylvanian Sage”—is a perennial endemic species of the Romanian flora, with a sporadic distribution [16]. Despite its phylogenetic similarity with recognized medicinal sages, particularly *S. pratensis*, which displays traditional uses for gastrointestinal, dermatological, and genitourinary conditions, *S. transsylvanica* is not formally recognized as a medicinal species in ethnobotanical sources [1,17,18]. At the same time, it was shown that its hydroethanolic extracts displayed low cytotoxicity against human normal gingival fibroblasts [19]. Previous phytochemical assessments of *S. transsylvanica* identified a phenolic profile broadly similar to *S. officinalis* and *S. glutinosa*, with rosmarinic acid and caffeic acid derivatives as dominant constituents and in vitro antioxidant and antimicrobial potential [10,19]. Recently, our team applied an untargeted phytochemical profiling and bioactive assessment of hydroethanolic extracts, with the results suggesting that this underexplored endemic species merits more rigorous investigations [20].

The extraction protocol has a crucial impact on the yield of bioactive metabolite recovery. Classical techniques (i.e., aqueous infusion and hydroethanolic maceration) remain widely used in ethnopharmacological practice and represent fundamental approaches for bioactivity comparisons; however, they are characterized by long extraction times, limited selectivity, and varying efficiency depending on compound polarity [21,22].

Ultrasound-assisted extraction (UAE) has emerged as a green alternative, exploiting acoustic cavitation to enhance solvent penetration, disruption of plant cell walls, and acceleration of mass transfer, thus resulting in shorter processing times and reduced solvent consumption [23,24,25]. Systematic optimization based on approaches such as response surface methodology (RSM) is currently used for the maximization of extraction efficiency with minimal resource or energy inputs [26,27,28]. Recent UAE optimization studies across the genus have been reported for *S. fruticosa*, [23], *S. deserta* [29], *S. nemorosa* [30], *S. miltiorrhiza* [31], *S. rosmarinus* [32], and *S. verticillata* [33]; however, no optimization study has considered *S. transsylvanica*, and no systematic comparison of extraction methods across its distinct plant organs has been reported.

Consequently, the present study aimed to: (a) develop and validate a D-optimal quadratic UAE optimization design for *S. transsylvanica* aerial parts, using total phenolic content and antioxidant capacity as response variables; (b) compare the optimized UAE protocol with classical aqueous infusion and 70% hydroethanolic maceration across three plant organs, aerial parts, flowers, and leaves, in terms of phytochemical yield and antioxidant activity; (c) characterize the phenolic and lipidic metabolite profiles of all extracts by targeted HPLC-MS analysis; and (d) evaluate the antimicrobial potential and assess all results using multivariate statistical analyses. To the best of our knowledge, this represents the first systematic extraction optimization applied to *S. transsylvanica*, contributing to the valorization of endemic Romanian flora as sources of bioactive compounds.

## 2. Materials and Methods

### 2.1. Reagents and Standards

The reagents necessary for spectrophotometric assays were purchased from Sigma-Aldrich Chemie GmbH (Schnelldorf, Germany). Reference standards for phenolic compounds were purchased from Sigma Aldrich, Carl Roth (Karlsruhe, Germany), Merck (Darmstadt, Germany), and Supelco (Bellefonte, PA, USA). HPLC-grade methanol and analytical-grade acetic acid were obtained from Merck KGaA (Darmstadt, Germany). Ultrapure water was obtained using a Milli-Q Direct 8 water purification system (Millipore, Bedford, MA, USA).

### 2.2. Identification and Harvesting

**Figure 1:**
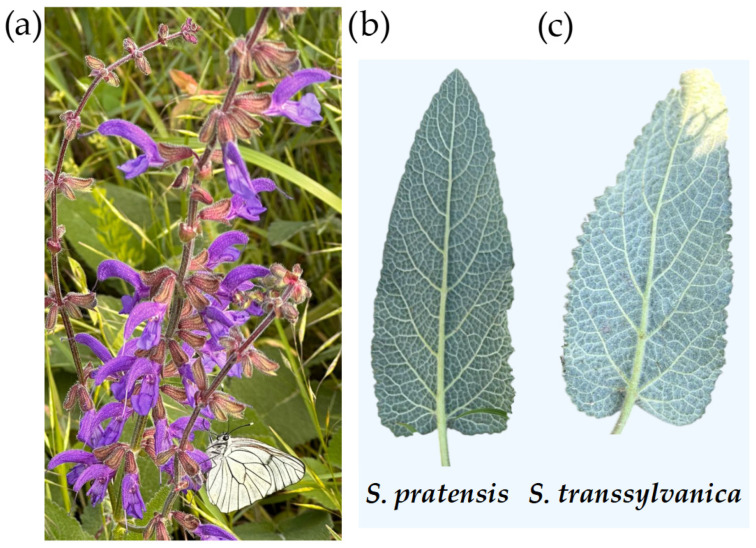
Identification of plant material: (**a**) *S. transsylvanica* aerial parts at peak flowering; differentiation from closely related species based on appearance on the abaxial (underside) leaf surface: (**b**) *S. pratensis* and (**c**) *S. transsylvanica*.

*S. transsylvanica* specimens (Figure 1a) were harvested from Băgău, Lopadea Nouă, Alba County, Romania (46°18′40.4″ N, 23°48′11.6″ E) without damaging the natural flora, during the flowering period (on 2 June 2023). Voucher specimens (both *S. transsylvanica* and *S. pratensis* for botanical comparison) were also deposited in herbarium of the Laboratory of Chromatography, Faculty of Horticulture and Business in Rural Development, University of Agricultural Sciences and Veterinary Medicine, Cluj-Napoca, Romania.

Differentiation from closely related *S. pratensis* and identification of *S. transsylvanica* were based on specialized Romanian flora (Figure 1b,c). As described by Ciocârlan in the *Illustrated Flora of Romania*, both species present calyx with short hairs and sometimes glandular, with ovate superior labium (upper lip) and ovate bracts. The following characteristics are therefore necessary for differentiation [16]:

- *S. transsylvanica* (Schur ex Griseb. & Schenk) Schur: pubescent-tomentose stem, with 3–5 pairs of leaves; the underside of the leaves is grayish-white colored and tomentose; perennial, sporadic, through sunny meadows, on eroded soils, from the forest-steppe zone to the oak forest zone;
- *S. pratensis* L. (Romanian vernacular name: “*salvie de câmp*”): stem with 1–3 pairs of leaves; short hairy on the upper part and inflorescence with glandular hairs; underside (abaxial surface) of the leaves is green, hairy on the veins; perennial, frequent at forest edges, scrublands, meadows, grassy cliffs, from forest-steppe to beech forest.

### 2.3. Treatment of Plant Material

Harvested specimens were directly subjected to classical drying inside the laboratory, without using an oven (on paper support, temperature of 20 ± 2 °C, protected from direct sunlight). After drying to a constant weight, the specimens were divided into three groups: *herba* (aerial parts or so-called “flowering tops”, consisting of leaves, stems, and flowers), *flos* (flowers, without separation from the upper portion of the stem), and *folium* (leaves). Lastly, the material was subjected to pulverization (1000 rpm, 1 min), using a laboratory knife mill (Grindomix GM 200, Retsch, Haan, Germany) and sieved for uniform granulometry (800 μm mesh size), and obtained pulverized material was kept sealed in paper bags in the dark, at temperatures of 20 ± 2 °C, until extraction.

### 2.4. Extraction Protocols

Classical extraction was applied according to previous studies of *Salvia* species from Romania, including *S. glutinosa*, *S. officinalis*, and *S. transsylvanica* [4,19]. Moreover, hydroethanolic ultrasound-assisted extraction (UAE) was applied as alternative “modern” extraction technique. The numbering used in sample notations refers to the plant organ: aerial parts/*herba* (1), flowers/*flos* (2), and leaves/*folium* (3). Extraction yields (expressed as percentages) were calculated based on the formula:

(1) $$Yield\;(\%\;dw)=100\times \left(\frac{mass\;of\;freeze-dried\;extract}{mass\;of\;dried\;plant\;material}\right)$$

#### 2.4.1. Classical Extraction

For infusion, powdered plant material was mixed with water at 100 °C (in a ratio of 1:100, *m*/*v*), and then the mixture was left to cool at room temperature for 30 min, with continuous stirring. Cooled down extracts were subjected to low-pressure paper filtration and freeze-dried, obtaining a dry infusion or aqueous extract (abbreviated **STA**).

For maceration, powdered plant material was mixed with 70% *v*/*v* hydroethanolic solution (in a ratio of 1:10, *m*/*v*), and then the mixture was kept in the absence of light at room temperature for a total of 10 days, with daily shaking. Cooled down extracts were subjected to low-pressure paper filtration, solvent evaporation, and freeze-dried, obtaining a dry ethanolic extract, macerate, or tincture (abbreviated **STE**).

#### 2.4.2. Ultrasound-Assisted Extraction

For UAE, powdered plant material was mixed (in a ratio of 1:10, *m*/*v*) with a given percentage of hydroethanolic solution (%, *v*/*v*) according to the experimental design, and then subjected to UAE using a SFX 150 Sonifier (Branson Ultrasonics Corporation, Brookfield, CT, USA) equipped with a tapered microtip (diameter of 3.2 mm), submersed at 2 cm in the extraction flask. The temperature of the extraction mixture was kept constant at ~0–5 °C, with the sample flask being immersed in an ice bath and under constant homogenizing with a magnetic stirrer (800 rpm). After each experimental run, the samples were centrifuged (16,000× *g*, 10 min), and visually clear supernatant was further subjected to low-pressure paper filtration. Obtained clarified samples were used for experimental design. Final samples, obtained using 38% hydroethanolic solution, were subjected to solvent evaporation (rotary evaporator, Heidolph Laborota 4000, Schwabach, Germany) and finally freeze-dried (Alpha 1-2 LDplus, Martin Christ Gefriertrocknungsanlagen GmbH, Osterode am Harz, Germany) to obtain a dry optimized extract (abbreviated **STO**).

### 2.5. Optimization of Hydroethanolic UAE

#### 2.5.1. Experimental Design

In order to explore the optimization of hydroethanolic UAE, a D-optimal experimental design (DoE) was developed using MODDE software version 12.0 (Sartorius, Göttingen, Germany) [34,35]. After confirming the nonlinear behavior of the system, a quadratic model was applied, and the significance of the quadratic terms were considered for each parameter. The generated DoE included an experimental matrix made up of three independent variables or factors, identified as relevant parameters for UAE: ultrasonic amplitude (%—later calculated as ultrasonic power, W), extraction time (min), and the concentration of hydroethanolic solution (%, *v*/*v*). Experimental runs included plant material from the *herba* category.

#### 2.5.2. Optimization Protocol

For the maximization of phytochemical yield based on given UAE parameters, the optimizer function of MODDE was used to maximize the recovery of phenolic metabolites with antioxidant potential. Additionally, the selection of potential optimal parameters was applied visually using the response surface plots and contour plots in each case [34].

### 2.6. Phytochemical Screening

Phytochemical screening was conducted using two in vitro methods: total phenolic content (TPC) based on Folin-Ciocalteu protocol, and total flavones and flavonols, frequently described as total flavonoid content (TFC), based on the simple aluminium chloride (AlCl_3_) protocol. The methods were adapted from previous protocols [4,19,36] to microplate reader (SPECTROstar, BMG Labtech, Ortenberg, Germany). Freeze-dried extracts were re-solubilized in the extraction solvent.

For TPC, 20 µL of samples (tested at 500 μg/mL) were mixed with 100 µL of diluted reagent (1:9, *v*/*v*) and after 3 min, 80 µL of Na_2_CO_3_ solution (7.5% in water) was added. The absorbance was read at λ = 760 nm after 30 min of incubation against solvent blank, with the results being expressed as milligrams of gallic acid equivalents (mg GAE/g dw), both for all extracts (for freeze-dried powder) as well as for the experimental runs of optimization (for dried plant material).

For TFC, 100 µL of samples (tested at 1 mg/mL) were mixed with 100 µL of AlCl_3_ solution (2% in water). The absorbance was read at λ = 420 nm after 10 min of incubation against sample blank, with the results being expressed as milligrams of quercetin equivalents per gram of freeze-dried powder (mg QE/g dw).

### 2.7. Antioxidant Capacity

Antioxidant capacity screening was applied using three in vitro methods: ABTS, DPPH (radical scavenging activity) and FRAP (ferric reducing antioxidant power). The methods were adapted from previous protocols [4,19,36] to microplate reader, and freeze-dried extracts were re-solubilized in the extraction solvent. All results were expressed as milligrams of Trolox equivalents per gram of freeze-dried powder (mg TE/g dw).

For ABTS, the radical aqueous solution was obtained from mixing 7 mM 2,2-azino-bis(3-ethylbenzothiazoline-6-sulfonic acid) (ABTS) solution with 2.45 mM potassium persulfate. 20 µL of samples (tested at 500 μg/mL) were mixed with 200 µL of radical solution, and the absorbance was read at λ = 734 nm after 6 min of incubation against reagent–solvent blank.

For DPPH, the radical solution was obtained from 1,1-diphenyl-2-picrylhydrazyl (0.004% in absolute methanol). 30 µL of samples (tested at 120–160 μg/mL) were mixed with 270 µL of radical solution, and the absorbance was read at λ = 515 nm after 30 min of incubation against reagent–solvent blank.

For FRAP, the reagent solution was obtained from acetate buffer (0.3 M, pH = 3.6), FeCl_3_ solution (20 mM in 40 mM HCl), and 2,4,6-tris(2-pyridyl)-*s*-triazine solution (10 mM in 40 mM HCl), in a 10:1:1 ratio (*v*/*v*/*v*). 25 µL of samples (tested at 120–160 μg/mL) were mixed with 175 µL of reagent solution, and the absorbance was read at λ = 593 nm after 30 min of incubation against solvent blank.

### 2.8. HPLC-MS Analysis of Phenolic Compounds

#### 2.8.1. HPLC-MS Protocol and Conditions

The analysis of phenolic compounds was performed using liquid chromatography–tandem mass spectrometry (LC-MS/MS) using a Nexera X3 HPLC system (Shimadzu Corporation, Kyoto, Japan) coupled to a QTOF 9030 mass spectrometer (Shimadzu). Samples were pretreated by centrifugation (Sigma 204 centrifuge, Osterode am Harz, Germany), weighing (Analytical Plus Balance, Mettler-Toledo, Greifensee, Switzerland), and ultrasonication (Elma Transsonic 700/H ultrasonic bath, Singen, Germany), to obtain clean analytical solutions suitable for chromatographic separation.

LC-MS/MS methodology was adapted from previously published protocols for the analysis of phenolic compound [37,38,39]. Separation was performed using reversed-phase chromatography with a Zorbax SB-C18 column (100 mm × 3.0 mm i.d., 3.5 μm particle size, Agilent Technologies), with mobile phase consisting of acetic acid in water (0.2% *v*/*v*, A) and acetic acid in methanol (0.2% *v*/*v*, B). To achieve gradient elution of A and B, the following scheme was followed: 0–30 min, linear gradient from 3% to 70% B; re-equilibration with 3% B for 4 min. HPLC parameters were: 1 mL/min flow rate, 48 °C column temperature, and 1 µL injection volume. For MS, detection was performed in negative ionization mode using either MS (SIM mode) or MS/MS (MRM mode), with details in Table S1 (Supplementary Materials). The electrospray ionization (ESI) source parameters were set as follows: source voltage −2000 V, interface temperature 300 °C, nebulizing gas flow 3 L/min, heating gas flow 15 L/min (air); drying gas flow 15 L/min (nitrogen), desolvation line temperature 300 °C, heat block temperature 400 °C.

#### 2.8.2. Quantification and Validation

Quantitative analysis was achieved by monitoring either the pseudo-molecular ion (in case of MS analysis) or specific fragmentation transitions in case of MS/MS analysis respectively, with details in Table S1. Data acquisition and processing were carried out using LabSolutions 5.128 software (Shimadzu Corporation, Kyoto, Japan) and the results were expressed as micrograms of phenolic compound per gram of dried extract (µg phenolic compound/g of dried extract) [38].

Validation of the analytical method was applied based on linearity, limit of detection (LOD), limit of quantification (LOQ), precision, and accuracy. Linearity was assessed in the 0.05–50 µg/mL range, LOD and LOQ were calculated based on the signal-to-noise ratio (S/N) of 5 and 10, respectively, and the calibration curve parameters are presented in Table S1 (Supplementary Materials).

### 2.9. LC-MS Analysis of Lipidic Metabolites

#### 2.9.1. Phytosterols

Phytosterol content was determined based on a LC-UV-MS/MS method, previously validated and used in other studies [38], using an 1100 HPLC system with UV detector and coupled to an Ion Trap 1100 SL mass spectrometer (Agilent Technologies, Santa Clara, CA, USA). Isocratic elution was applied using acetonitrile: methanol (90:10, *v*/*v*) as mobile phase, at a flow rate of 1 mL/min, temperature of 45 °C, and 10 μL of injected solution. For MS, positive ionization by APCI was used, coupled with an Ion Trap. Working parameters were adjusted to nitrogen gas temperature 325 °C, flow rate 7 L/min, nebulizer pressure 60 psi, capillary voltage 4000 V. Data acquisition and interpretation were achieved using DataAnalysis (v5.3) and ChemStation (vB01.03) software from Agilent Technologies (Santa Clara, CA, USA). Analytical standards were injected in the same conditions (for quantification and MRM): brassicasterol, campesterol, ergosterol, stigmasterol, and beta-sitosterol. Results were expressed as nanograms of phytosterol per gram of dried extract (ng/g of dried extract).

#### 2.9.2. Tocopherols

Tocopherol content was determined based on a LC-MS/MS method, previously validated and used in other studies [38], using the same equipment as described in Section 2.9.1. In this case, a Zorbax SB-C18 column (100 mm × 3.0 mm i.d., 3.5 µm) was used. Isocratic elution was applied using water: methanol (7:93; *v*/*v*) as mobile phase, at a flow rate of 1 mL/min, temperature of 40 °C, and 10 μL of injected solution. For MS, positive ionization by APCI was used, coupled with an Ion Trap. Analytical standards of alpha/gamma/delta-tocopherol were injected in the same conditions. Results were expressed as nanograms of tocopherol per gram of dried extract (ng/g of dried extract).

### 2.10. Antimicrobial Activity

The antibacterial and antifungal potential was determined using the disk diffusion method and the microdilution method, based on standard protocols from the literature [40,41]. Initial microbial strains were selected based on their potential to produce gastrointestinal infections, as follows: *Staphylococcus aureus* (ATCC 25923), *Enterococcus faecalis* (ATCC 29212), *Bacillus cereus* (ATCC 11778), *Escherichia coli* (ATCC 25922), *Pseudomonas aeruginosa* (ATCC 27853), and *Candida albicans* (ATCC 10231). The freeze-dried extracts were resolubilized in 10% DMSO in water solution at a concentration of 100 mg/mL and then subjected to the analyses.

#### 2.10.1. Diffusion Method

For the Kirby-Bauer disk diffusion test, each microbial suspension was inoculated on Petri dishes containing Mueller-Hinton agar (for bacteria) and Sabouraud dextrose agar (for *Candida*), after adjustment of inoculum suspension concentration to 0.5 McFarland, and then the agars were left to dry (20 min, 35 °C). Aseptic wells (6 mm diameter) were made and then 50 µL of extract was added to each well. Positive controls were chosen according to strain sensibility, using amoxicillin (30 μg), ciprofloxacin (10 μg), amikacin (10 μg), and ketoconazole (10 μg) disks, while negative control was 10% DMSO. Subsequently, the plates were incubated at 37 °C for 24 h (bacteria), and at 28 °C for 48 h (fungi). The inhibition zone diameters (mm) were measured for triplicate measurements (*n* = 3), for both positive and negative controls.

#### 2.10.2. Determination of the Minimum Inhibitory Concentrations

The minimum inhibitory concentration (MIC) values were determined using a serial microdilution technique for the extracts showing antimicrobial activity [42]. Bacterial suspensions were adjusted to approximately 10^6^ CFU/mL in Mueller–Hinton broth, in sterile 96-well microplates, along with positive controls (without extracts) and sample controls (without bacteria). Then, 10 µL of each bacterial suspension was mixed with 200 µL decreasing concentrations of extract, diluted using broth (expressed as 25%, 10%, 5%, 2.5%, and 1.25% *v*/*v* from 100 mg/mL). After incubation at 37 °C for 24 h, optical density was read at λ = 600 nm on a BioTek Synergy 2 multichannel spectrophotometer (BioTek Instruments, Winooski, VT, USA). The MIC was defined as having the lowest concentration presenting a complete inhibition of microbial growth. The analysis was performed in triplicate.

### 2.11. Statistical Analysis and Software

Primary statistical analysis was applied using MODDE Sartorius and GraphPad Prism 8. Each determination was applied in triple replicate and expressed as average value ± standard deviation (*n* = 3). Statistical difference was determined using one-way ANOVA with post-hoc analysis (Tukey’s test), where *p* values < 0.05 represented statistically significant differences. Summary of fit (threshold values R2 > 0.50, Q2 > 0.50, validity > 0.25, and reproducibility > 0.50) and ANOVA lack of fit and regression tests (threshold for F and *p* values < 0.05) regarding DoE and optimization were analyzed in MODDE.

Multivariate statistical analysis was applied using R Studio version 2026.01 (Posit PBC), through the generation of heatmap with hierarchical clustering (Ward’s minimum variance—Ward.D2, correlation-based distance; standardized z-score-transformed variables) and principal component analysis (PCA) biplot (Pareto-scaled variables), employing the pheatmap [43], FactoMineR, ggplot2, and ggrepel packages [44].

## 3. Results and Discussion

### 3.1. Experimental Design and Summary of Fit

The experimental design included an experimental matrix (presented in Table 1) based on three independent variables (using three factor levels), denoted as ultrasonic amplitude (%, X_1_), ethanol concentration (% *v*/*v*, X_2_), extractive time (min, X_3_). In order to determine their effects during the UAE protocol, TPC, ABTS, and DPPH were determined, serving as dependent variables (Y_1–3_). As can be observed, the assessed values showed high variability, namely 15.00–45.78 mg GAE/g (TPC), 37.40–87.24 mg TE/g (ABTS), and 44.90–127.92 mg TE/g (DPPH).

The summary of fit is a statistical assessment tool evaluating the performance of the experimental model using four main parameters (where values = 1.0 correspond to a perfect model). R2, indicating model fit, presented high values (0.99 for TPC and 0.97 for both ABTS and DPPH), while Q2 values estimated a model with acceptable prediction precision (0.92 for TPC, 0.78 for ABTS, and 0.81 for DPPH). Model’s validity was indicated by high values (0.86 for TPC, 0.74 for ABTS, and 0.82 for DPPH) and demonstrated good reproducibility based on the variability of replicates (0.97 for TPC, 0.96 for ABTS, and 0.94 for DPPH). Furthermore, summary of the regression and residual analysis using ANOVA revealed that the models adequately fit the experimental data, with lack of fit *p* values of 0.734 (TPC), 0.359 (ABTS), and 0.496 (DPPH). Consistent with prior experimental models [26,35], the fit summary validated the experimental design, justifying continuation of the analyses.

### 3.2. Influence of Extraction Variables

**Figure 2:**
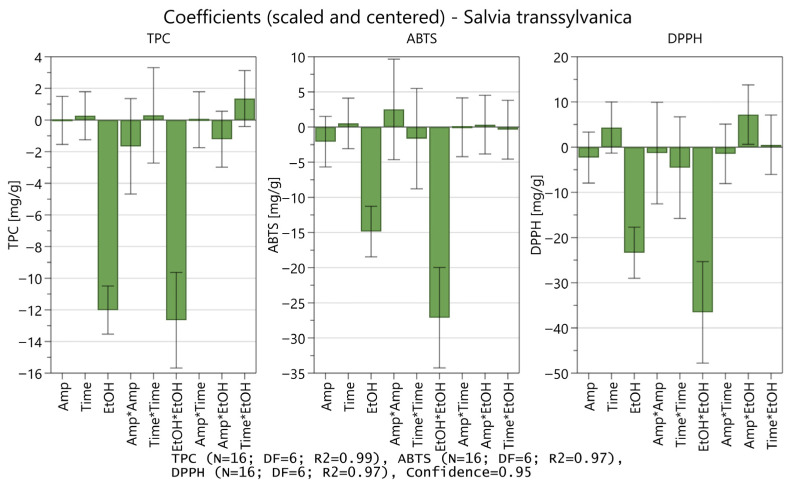
Coefficient plots (scaled and centered data) for each extraction variable, TPC, ABTS, and DPPH, including both significant and insignificant terms (main effects, interactions, and quadratic). * represents multiplication sign.

To assess the influence of extraction variables on the extractive yields, the coefficient plots were analyzed for each dependent variable (Y_1–3_—TPC, ABTS, and DPPH). As deduced from the coefficient plots (Figure 2), the impact of extraction parameters presented a similar pattern. Namely, for all three parameters Y_1–3_, the hydroethanolic concentration (EtOH) and its quadratic influence (EtOH × EtOH) were significant, with *p* values < 0.05. Moreover, for DPPH the interaction of amplitude and hydroethanolic concentration (Amp × EtOH) was also significant (*p* = 0.03).

To analyze these observations in greater detail, contour plots were generated for each dependent variable.

**Figure 3:**
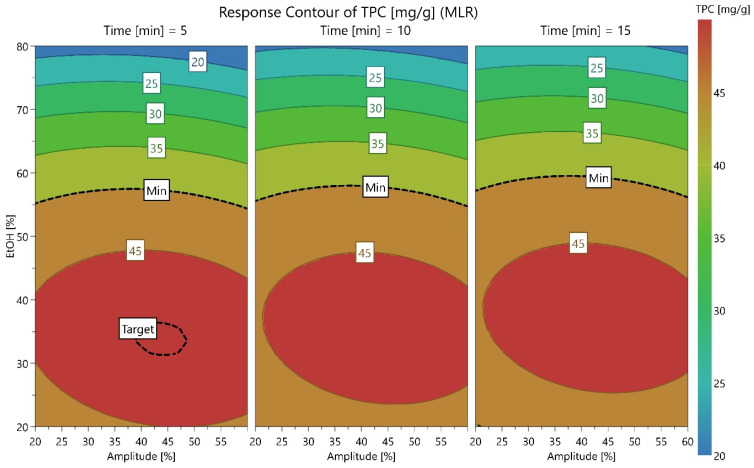
Contour plot for TPC, expressed as mg GAE/g, showing the variation according to amplitude (%), ethanol concentration (%), and three levels of time (5, 10, and 15 min).

In the case of TPC (Figure 3), lowering the EtOH concentration from 80% to approximately 35% induced a significant rise in the yields, from 15 to over 45 mg GAE/g. Ultrasonic amplitude and time were less influential, while the circular pattern of amplitude indicated a maximal TPC yield at values closer to 40%, with maximal values for ~35% ethanol.

**Figure 4:**
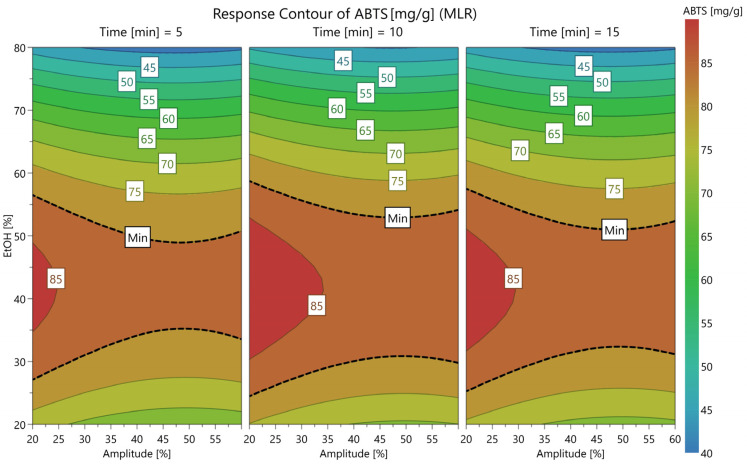
Contour plot for ABTS, expressed as mg TE/g, showing the variation according to amplitude (%), ethanol concentration (%), and three levels of time (5, 10, and 15 min).

Similarly, for ABTS (Figure 4), lowering the EtOH concentration from 80% to approximately 45% induced a significant rise in antioxidant potential, from 40 to over 85 mg TE/g. The effect of amplitude was particularly evident at lower values, where amplitudes under 30% were necessary to achieve maximal ABTS values, regardless of the extraction time.

**Figure 5:**
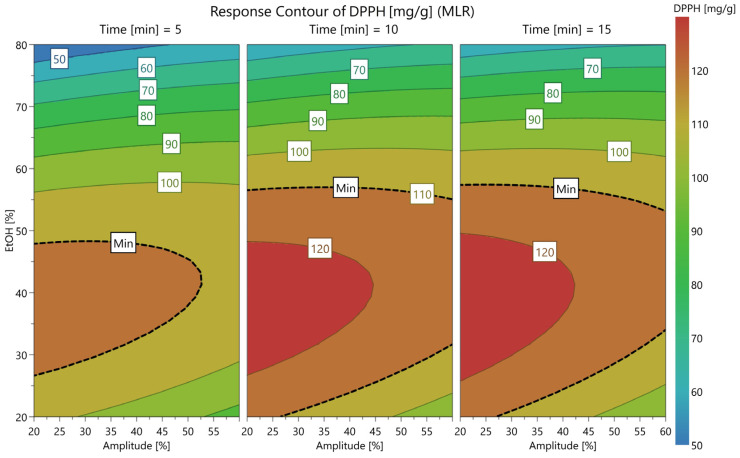
Contour plot for DPPH, expressed as mg TE/g, showing the variation according to amplitude (%), ethanol concentration (%), and three levels of time (5, 10, and 15 min).

Correspondingly, for DPPH (Figure 5), lowering the EtOH concentration from 80% to approximately 40% induced a significant rise in the yield of antioxidant metabolites, from 45 to over 120 mg TE/g. Amplitude showed a moderate influence at lower EtOH concentrations, where values under 40% were necessary for the achievement of maximal DPPH values, considering time intervals of 10–15 min.

As observed, phenolic yields declined at ethanol concentrations above 40%, which can be mainly attributed to a polarity imbalance between the solvent and the predominantly polar phenolic species, typically exhibiting reduced solubility in less-polar solvent mixtures. As such, low water content and high ethanol concentrations lower solvent polarity below the limits of efficient solvation. This observation was previously noted for *S. officinalis*, where 30% ethanol concentrations were optimal for total phenolics [45], and for *S. fruticosa*, where 68% ethanol improved phenolic yield during UAE [23]. Additionally, the contribution of increased viscosity at intermediate ethanol concentrations, as a limitation for mass transfer, has also been reported for similar extraction systems [21,46].

The secondary role of ultrasonic amplitude and extraction time in the present study can be attributed to the use of mildly pulverized plant material. Specifically, when the plant matrix is mechanically treated, giving rise to reduced particle sizes prior to sonication, cavitation does not provide additional substantial physical disruption of intact cell walls [47,48]. Thus, it is plausible that the dominant determinant of extraction yield shifts toward solvent polarity [49]. This interpretation is consistent with recent observations on *Cornus mas*, in which amplitude converged to the lowest tested value (20%) at the optimum [50]. Furthermore, although the delivered ultrasonic power across the amplitude range tested in this study was low in absolute terms (~4.3–15.6 W applied for 25.0–29.34 mL solvent, corresponding to approximately 0.15–0.60 W/mL), it was presumably sufficient to enhance mass transfer without producing the degradation of heat-sensitive phenolics.

### 3.3. Optimization and Validation of Hydroethanolic UAE

**Figure 6:**
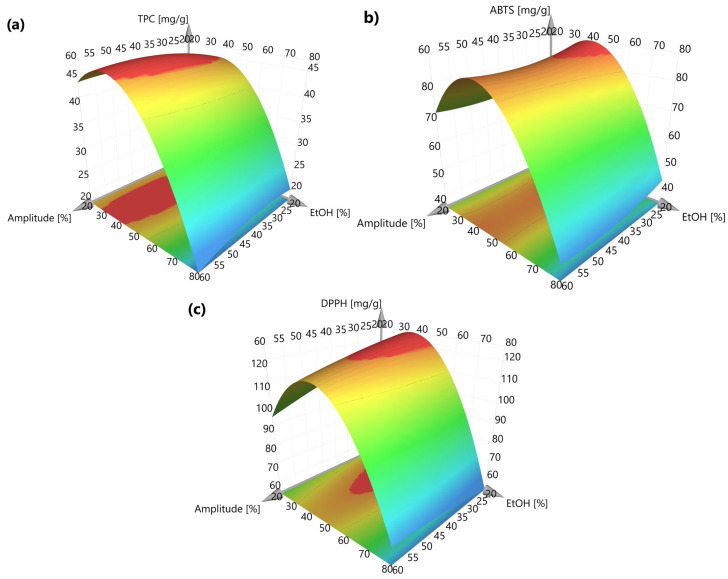
Response surface plots (RSP) for EtOH concentration and amplitude as independent variables, with time being constant at 12 min. Each RSP is represented for dependent variables of the model, namely (**a**) TPC and in vitro antioxidant potential using (**b**) ABTS and (**c**) DPPH assays.

While ultrasonic amplitude and extraction time exerted a minor influence on extractive yields, with moderate effects observed only for the 30–50% EtOH range, hydroethanolic concentration emerged as the key factor governing the recovery of phenolic compounds with antioxidant potential. The nonlinear behavior of the model, evidenced by the significant quadratic term EtOH × EtOH (*p* < 0.001), induced a marked improvement in model fit and is readily apparent in the curvature (“plateau surface”) of the response surface plots (RSP, Figure 6) [27].

After studying the influences of extraction parameters and validation of the model, attention was focused on finding an optimal combination of independent variables which can induce a maximal yield in phenolic metabolites with antioxidant potential. The optimizer function in MODDE identified the values as 24% ultrasonic amplitude (corresponding to ~5 W), 12 min extraction time, and 38% EtOH concentration. Using these parameters, an optimal extract was developed, showing high experimental relative recoveries (105–119%) in comparison to theoretically predicted values (Table 2). The consistently positive recoveries suggest that the quadratic model slightly underestimates yields at the optimum, a behavior that can be observed in the case of polynomial RSM models with optimal points near the boundary of the experimental domain [27]. Nevertheless, recovery values fall within the 100 ± 20% validation range, confirming the predictive adequacy of the model [23].

The optimization of classical and UAE parameters has been addressed in several studies regarding *Salvia* species, with RSM representing one of the most frequently employed frameworks. For *S. fruticosa*, a UAE was conducted on post-distillation residues, with TPC and antioxidant activity as response variables, identifying 68% ethanol as the optimal solvent concentration for phenolic recovery, quantified as 191.77 mg GAE/g [23]. In the case of *S. deserta* flowers, UAE of polyphenols was optimized using both RSM and deep neural network approaches simultaneously, demonstrating that 55% ethanol was the most efficient for the extraction of caffeic acid, protocatechuic acid, and rosmarinic acid [29].

At the same time, ultrasonic pretreatment conditions were assessed for *S. nemorosa* as influential for TPC yield and antioxidant capacity [30], while for *S. miltiorrhiza* simultaneous RSM and artificial neural network optimization was applied to maximize the yields of two structurally distinct target classes: lipophilic tanshinone IIA and polar salvianolic acid B [31]. For *S. rosmarinus*, UAE applied with 70–90% ethanolic solvent mixtures was explored for the optimal recovery of ursolic, oleanoic, and rosmarinic acids [51], while the supercritical extraction of rosmarinic acid was optimized using *S. sclarea* distillation residues [52]. Lastly, RSM-based UAE optimization of total phenolic compounds from *S. verticillata* roots has demonstrated the applicability of the approach beyond aerial plant organs [33].

Finally, the optimal parameters were applied for all three types of plant material, with the development of optimized extracts STO1–3. Then, these extracts were compared with classical extracts (STA1–3 and STE1–3).

### 3.4. Extraction Yields

The extraction yield was determined gravimetrically and expressed as the percentage of freeze-dried extract recovered per unit of dried plant material. The results varied significantly across extraction protocols and helped to interpret phytochemical data (Table S2) in alternative ways. Aqueous infusions (STA) yielded the highest dry extract mass across all plant organs, ranging from 24.2% (STA2, flowers) to 28.9% (STA3, leaves). Next, hydroethanolic macerates (STE) produced intermediate yields of 17.3–19.4%, while the optimized UAE extracts (STO) consistently showed the lowest yields, ranging from 11.1 to 11.7% dw, which represented approximately half the yield of the infusions and two-thirds of the macerates.

This descending order in the yields (infusion > maceration > optimized UAE) may be a result of solvent selectivity. During infusion, the contact with water at 100 °C, followed by a decrease in temperature over 30 min, is not selective for phenolic compounds, but rather dissolves several cellular constituents, including polysaccharides (arabinogalactans, pectins), monomer sugars, and organic acids [53]; as such, a rather high mass is recovered in the detriment of the phenolic fraction. On the other hand, lower polarity obtained using 70% hydroethanolic maceration results in a more selective phenolic fraction [54], excluding highly polar macromolecular constituents. Lastly, the most selective gravimetric yield of the UAE protocol, based on 38% ethanol and 12 min of contact, suggested that a moderate polar solvent mixture produced the highest phenolic enrichment.

These observations regarding solvent selectivity have practical consequences when bioactive effects and quantified metabolites are re-calculated based on a plant-equivalent basis (multiplication by yield; see also Section 3.5, Section 3.6 and Section 3.7). As an example, STA1 delivered approximately 169.55 × 0.2543 = 43.12 mg GAE per gram of dry plant material, in comparison to 162.80 × 0.1728 = 28.13 mg GAE/g for STE1 and 230.62 × 0.1171 = 27.01 mg GAE/g for STO1. It is worth noting that these observations do not undermine the value of the optimized UAE, but interpretation of the results needs to be correctly framed. As mentioned, the advantages of UAE over classical approaches stem from the shorter processing time (12 min versus 30 min and 10 days), lower thermal exposure (for the preservation of thermolabile metabolites), and the production of more concentrated and compositionally selective extracts.

### 3.5. Phytochemical Screening

**Figure 7:**
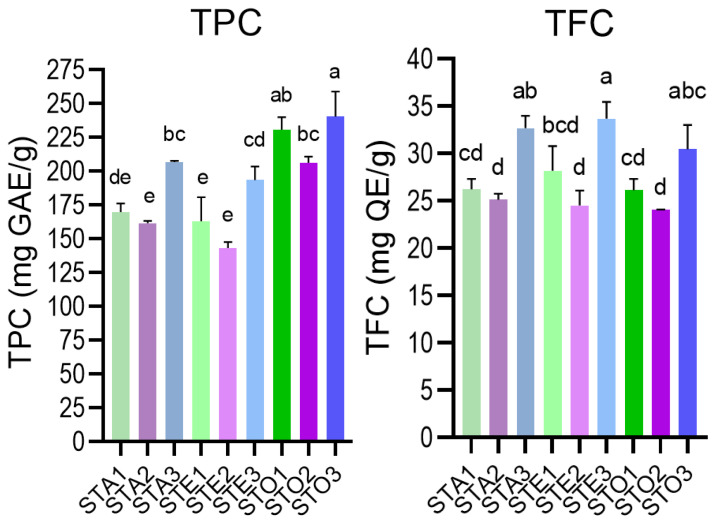
Graphical comparison of phytochemical screening (TPC—total phenolic content; TFC—total flavones and flavonols using AlCl_3_ method) results for ST (*S. transsylvanica*) extracts: A—aqueous, E—hydroethanolic; O—optimized UAE. Plant material: 1—aerial parts; 2—flowers; 3—leaves. Different superscript letters represent significant statistical differences between values for the same determination (one-way ANOVA, post-hoc Tukey’s test, *p* < 0.05).

Phytochemical screening was applied to all extracts before HPLC-MS, using in vitro assays for total phenolic (TPC) and flavonoid content (TFC)—specifically, the Folin-Ciocâlteu method and the AlCl_3_ method with specificity for flavones and flavonols. The results are given in Table S2, as well as in Figure 7.

The high TPC and TFC values indicated the phenolic richness of all *S. transsylvanica* extracts. Optimized UAE extracts (STO1–3) consistently showed the highest TPC values for each plant organ (230–240 mg GAE/g) and the leaf extracts exceeded the corresponding values for infusions and macerates (*p* < 0.02). In contrast, TFC values (24.05–33.68 mg QE/g) were not significantly different (*p* > 0.05) among different extract protocols and suggested a significant contribution of flavones and flavonols to total phenolic fraction [36]. Organ-specific differences were observed at the same time, where leaf extracts contained higher TPC and TFC values in comparison to flowers and aerial parts extracts.

The TPC and TFC values recorded also confirm *S. transsylvanica* as a particularly rich source of phenolic compounds relative to the published *Salvia* literature. The observed TPC values are higher than those reported for *S. nemorosa*, where 70 wild populations from western Iran ranged from 33.87 to 66.24 mg GAE/g dry weight [55], and is at the upper boundary of the broad range documented for *S. officinalis* methanolic extracts (80–150 mg GAE/g) [56]. The TFC values are likewise elevated, consistent with the dominant contribution of luteolin and apigenin glycosides (flavones) to which the AlCl_3_ assay is particularly sensitive [36].

### 3.6. Antioxidant Activity

**Figure 8:**
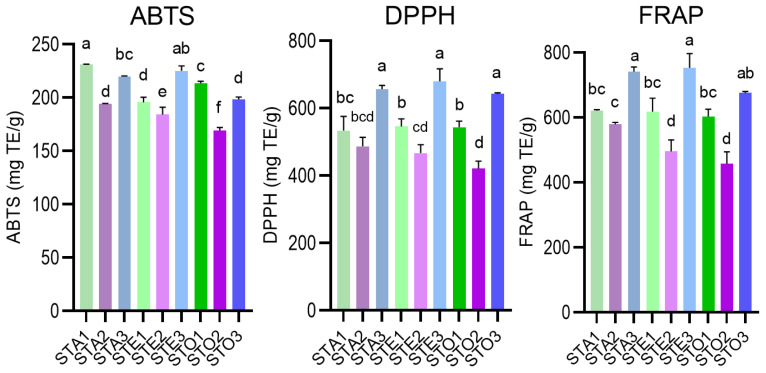
Graphical comparison of antioxidant potential for ST (*S. transsylvanica*) extracts: A—aqueous, E—hydroethanolic; O—optimized UAE. Plant material: 1—aerial parts; 2—flowers; 3—leaves. Different superscript letters represent significant statistical differences between values for the same determination (one-way ANOVA, post-hoc Tukey’s test, *p* < 0.05).

The antioxidant potential of the extracts was assessed using three complementary assays, ABTS, DPPH, and FRAP, each capturing different aspects of radical-scavenging and ferric reducing capacity. The results are presented in Table S2 and illustrated in Figure 8. Across all determinations, the observed values were uniformly high, confirming *S. transsylvanica* as a potent antioxidant plant material consistent with its phylogenetic placement within the *Salvia* genus.

A consistent divergence was observed between the ABTS assay on the one hand, and the DPPH and FRAP assays on the other, with respect to which extract type ranked highest. For ABTS, the aqueous infusion of aerial parts (STA1, 231.02 mg TE/g) achieved the highest value, and infusions generally performed comparably to or above the corresponding hydroethanolic macerates and UAE extracts. In contrast, for DPPH and FRAP, the leaf extracts consistently achieved the highest values, statistically exceeding most flower and aerial-part extracts (*p* < 0.01); at the same time, there was no significant difference between the leaf extracts (*p* = 0.06–1.00).

Regarding organ-specific effects, leaf extracts outperformed flower and aerial-part extracts for DPPH and FRAP across all three extraction methods (with maximal values for STE3, 679.66 and 752.87 mg TE/g, respectively), while flower extracts displayed the lowest values for most assays, with STE2 and STO2 frequently forming a single statistical group at the lower end of the spectrum. These observations were consistent with the TPC and TFC results (Section 3.5) and with HPLC-MS quantification showing the highest concentrations of rosmarinic acid and flavone glycosides in leaf material (Section 3.7).

A notable finding is that, apart from some flower extracts (STA2 and STO2), the three protocols generally yielded extracts with statistically indistinguishable antioxidant capacity (*p* > 0.05 for most pairwise comparisons). For ABTS in particular, STO2 (169.31 mg TE/g dw) was significantly lower than STA1 and STE3, suggesting that the moderate-polarity UAE solvent (38% EtOH) is less effective than either pure water or 70% ethanol for extracting the water-soluble flavonoid fraction from flower material specifically.

The antioxidant values substantially exceed those reported in the only prior study on this species, where 70% hydroethanolic extracts displayed antioxidant potential expressed as 59.29 (DPPH), 77.35 (ABTS), and 90.94 mg TE/g (FRAP) [19]. For other Romanian *Salvia* species studied under comparable protocols, the present results were comparable in range to those reported for *S. glutinosa* infusions and macerates by Nicolescu et al., with values as high as 422.32 (ABTS, infusions) and 737.41 mg TE/g (FRAP, macerates) [4].

### 3.7. HPLC-MS Analysis of Phenolic Metabolites

HPLC-MS separation and identification of major phenolic compounds was applied based on a targeted approach, considering frequently encountered metabolites from *Salvia* species and our previous study [20], in which several phenolic acids (rosmarinic acid, salvianolic acid K isomers) and flavonoids (luteolin and apigenin hexosides) were identified. In this study, 38 metabolites were identified in total, from which 23 were phenolic acids and related derivatives (such as glycosides and aldehydes), 13 were flavonoids and flavonoid-glycosides, and 2 were furanocoumarins with phenol moieties. Overall, the phenolic profile was dominated by caffeic acid derivatives, particularly rosmarinic acid and its methyl ester, together with salvianolic acid K, alongside flavone aglycones and glycosides of luteolin and apigenin.

#### 3.7.1. Phenolic Acids

Targeted LC-MS analysis (Table 3) confirmed rosmarinic acid as the major phenolic component of all *S. transsylvanica* extracts (representing ~90% of all phenolic acids), similarly to previous determinations on *Salvia* species [19]. This metabolite reached approximately 16–19 mg/g dry extract in both infusions and macerates, with only slightly lower values in the optimized UAE samples (11.9–13.5 mg/g). Among the tested extracts, flowers (STA2, STE2) and aerial parts (STA1, STE1) yielded the highest rosmarinic acid quantities.

Methyl rosmarinate and salvianolic acid isomer K were following in abundance, occurring in the approximate range of 400–900 and 100–900 µg/g, respectively, with the highest values generally observed in leaf and flower macerates, followed by infusions and, finally, UAE extracts. For both metabolites, the STE3 extract achieved maximal recoveries, with values of 860 and 887 µg/g. This distribution suggests that prolonged contact with 70% ethanol favored the extraction of rosmarinic acid derivatives, while intermediate-polarity UAE conditions privileged monomer phenolic acids.

Among simple hydroxycinnamic acids, caffeic and ferulic acids were consistently detected in all extracts, with a clear enrichment trend of STO > STE > STA for caffeic acid and of STE and STO > STA for ferulic acid. Caffeic and ferulic acids showed maximal recoveries after UAE, with values as high as 632 µg caffeic acid/g and 250 µg ferulic acid/g for the STO2 sample. The quantities of caffeic acid were then followed by tinctures (maximal 363 µg/g for STE2) and infusions (maximal 170 µg/g for STA2), a tendency also observed for ferulic acid.

Other phenolic acids were detected in smaller concentrations and influenced by distinct extraction patterns. For example, 3,4-dihydroxyphenyllactic acid (also known as danshensu or salvianic acid A) was detected with values up to 150 µg/g (STE3), along with its *O*-glucoside. Some other benzoic and hydroxycinnamic acids, as well as their glycosides (e.g., protocatechuic acid and protocatechuic acid-4-*O*-glucoside, vanillic and syringic acids and their 4-*O*-glucosides, 3-hydroxybenzoic, salicylic, and *p*-coumaric acids), were present at lower levels (generally in the range of 10–100 µg/g). In several cases, macerates yielded the highest concentrations (e.g., *p*-coumaric and protocatechuic acid), whereas UAE afforded intermediate recoveries.

Taken together, the phenolic acid profile of *S. transsylvanica* extracts is consistent with the chemotaxonomic signature identified across *Salvia* species, with caffeic acid and its oligomers, rosmarinic acid in particular, constituting the dominant phenolic fraction [7,8,57]. The rosmarinic acid content is comparable to or slightly lower than the highest values reported for other optimized UAE extracts. For example, previous studies showed recoveries up to 20 mg/g in *S. officinalis* [58], *S. rosmarinus* [32,59], and *S. sclarea* [60], although the differences in expression (dried extract vs. dried plant material) makes absolute numerical comparison difficult. Regardless, *S. transsylvanica* can also be regarded as rosmarinic acid-rich species.

At the same time, the co-occurrence of methyl rosmarinate (as rosmarinate ester) and salvianolic acid K (as caffeic acid trimer), both reaching up to ~900 µg/g in leaf macerates, is consistent with observations in *S. officinalis* extracts produced under analogous protocols; for example, Dent et al. quantified ~5 mg/100 g methyl rosmarinate and ~150 mg/100 g salvianolic acid K using 30% ethanol under high hydrostatic pressure [45]. In contrast, the systematic enrichment of caffeic and ferulic acid monomers in UAE extracts highlight a complementary influence of ultrasonic effects (i.e., acoustic cavitation) on cell wall disruption and metabolite release [28].

The consistently high cumulative phenolic acid (CPA) content across all three extraction protocols (ranging from 13.5 to 20.9 mg/g dry extract; decreasing in the order STE ≳ STA > STO) underlines the richness of the phenolic profile in *S. transsylvanica*.

#### 3.7.2. Flavones, Flavonols, and Coumarins

The flavonoid fraction (Table 4) was quantitatively less abundant than phenolic acids but qualitatively diverse, comprising apigenin and luteolin aglycones, their acylated glucosides, as well as several flavonol derivatives. Apigenin-7-*O*-glucoside and luteolin-7-*O*-glucoside were the most abundant, reaching up to ~2.1 mg/g (STE2) and ~1.1 mg/g (STE3), respectively. Luteolin-7-*O*-(6″-*O*-malonyl-glucoside) also accumulated substantially, with high levels in leaf extracts (maximal 454 µg/g in STO3).

Flavone aglycones, belonging to both non-methoxylated (apigenin, luteolin) and methoxylated classes (eupatorin, genkwanin, and hispidulin), occurred mainly in hydroethanolic macerates and UAE extracts, with much lower levels in infusions. This distribution reflects the lower polarity of aglycones and the ability of ethanolic systems to more efficiently desorb and solubilize them from cellular components. In contrast, glycosylated flavonols (hyperoside, isoquercitrin) and coumarins (esculin, esculetin) were detected in all extract types, but with a moderate preference for tinctures and optimized UAE.

Overall, the flavonoid fraction of *S. transsylvanica* adheres to the characteristic pattern described for the genus, where luteolin and apigenin glycosides represent the dominant flavone constituents and flavonol derivatives (isoquercitrin, hyperoside) are present as minor components [7,61]. The cumulative flavonoid content (CFL, ranging from 1113 to 3770 µg/g) is substantially lower than CPA but is nonetheless comparable to reported values for other *Salvia* species studied under equivalent hydroethanolic conditions.

The presence of methoxylated flavone aglycones (eupatorin, genkwanin, hispidulin) predominantly in hydroethanolic and UAE extracts, together with the consistent detection of the coumarin glycoside esculin and its aglycone esculetin across all extract types, further expands the phytochemical diversity of *S. transsylvanica*. Although less frequent, coumarin derivatives (such as esculetin) were identified in other sage species, such as *S. pinnata* [62].

### 3.8. HPLC-MS Analysis of Lipidic Metabolites

To further assess the extractability of lipidic metabolites using classical and optimized hydroethanolic protocols, two main classes were analyzed using HPLC-MS (Table 5). Due to their affinity for less-polar solvents, lipidic metabolites were not assessed in aqueous extracts. Classical hydroethanolic macerates (STE) were superior to optimized extracts (STO) regarding phytosterols and tocopherols.

From the phytosterol class, beta-sitosterol was identified in the highest concentration in macerates (maximal 6.99 mg/g in the flower extract). Similarly, brassicasterol was the most abundant in the flower extract (0.94 mg/g), with slightly lower values of stigmasterol. For UAE, the sterol concentrations were negligible, while campesterol levels were modest in all extracts. A similar pattern was observed for tocopherols, which were only detected in tinctures in low quantities, specifically as gamma-tocopherol (3.69 μg/g for STE3) and as delta-tocopherol (0.873 μg/g for STE2). Tocopherols were not detected in the optimized extracts.

This clear protocol differentiation is explained by solvent polarity and extraction time, where optimized extracts used more polar (38% ethanol) and short sonication time, while tinctures used less polar (70% ethanol) and longer extraction times (10 days). As such, the conditions employed during UAE are suboptimal for hydrophobic sterols and tocopherols [22]. The literature data for these metabolites is scarce; alpha-tocopherol recovery from the leaves of 60 *Salvia* species was also previously achieved using absolute methanol, achieving values as high as 972 µg/g for *S. nubicola* [63].

### 3.9. Antimicrobial Potential

**Figure 9:**
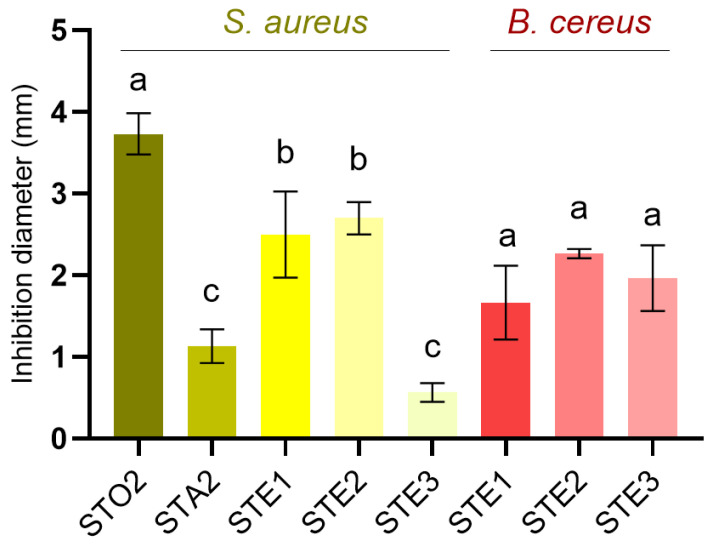
Determination of antibacterial potential using Kirby-Bauer disk diffusion test, against *S. aureus* (left) and *B. cereus* (right). Values are displayed as average ± standard deviation (*n* = 3). Values for the positive controls: 14.60 ± 1.53 mm (amoxicillin 30), 11.00 ± 1.00 mm (amikacin 10). Different superscript letters represent significant statistical differences between values for the same determination (one-way ANOVA with post-hoc analysis using Tukey’s test; *p* < 0.05).

The antimicrobial potential of *S. transsylvanica* extracts was assessed against a panel of microbial, selected to reflect the gastrointestinal infectious context associated with the traditional use of sage preparations [1]. Of the five strains, measurable inhibition zones were recorded only against Gram-positive bacteria *S. aureus* and *B. cereus* (Figure 9 and Figure S1), while no activity was detected against the Gram-negative species *E. coli* and *P. aeruginosa* or the fungal strain *C. albicans* at the tested concentration of 100 mg/mL.

For *S. aureus*, inhibition zone diameters ranged from 0.5 to 4 mm across the nine extracts, with STO2 yielding the largest zone of 3.73 ± 0.25 mm and being followed by STE1 and STE2. For *B. cereus*, inhibition zones ranged from 1.2 to 2.3 mm only for the hydroethanolic extracts, without any significant difference (*p* > 0.05). This indicates that the flower extracts displayed the highest antibacterial activity, yet the observed effects should be regarded mainly as moderate and selective against *S. aureus*.

The application of serial microdilution confirmed these findings, with selective MIC values of 3.12 and 6.25 mg/mL against *S. aureus* for the most active extracts (STE1–2, STA2, and STO2).

The selective activity against Gram-positive bacteria and the absence of inhibition against Gram-negative strains is consistent with patterns widely reported for *Salvia* essential oils and polyphenolic-enriched extracts [5,64,65]. Moreover, the antimicrobial activity observed in this study agrees with the preliminary phytochemical assessment of *S. transsylvanica* by Mocan et al. [19], who reported inhibitory activity of hydroethanolic extracts against Gram-positive strains, including *S. aureus*. Similar selective Gram-positive activity has been documented for other Romanian *Salvia* species: Veličković et al. reported inhibitory activity of *S. glutinosa*, *S. officinalis* and *S. pratensis* ethanol extracts against *S. aureus* and *B. subtilis* [66,67], while Bălășoiu et al. confirmed antibacterial potential of *S. glutinosa* and *S. verticillata*, predominantly directed at Gram-positive strains [68]. These effects, although modest at the tested concentrations, place *S. transsylvanica* within the expected antimicrobial profile of the genus, rather than identifying it as exceptional in this regard.

Reinforcing results of phytochemical composition, the most active extracts accumulated the highest concentrations in caffeic/ferulic acids (STO2) and apigenin-7-*O*-glucoside (STE1, STE2, and STA2), highlighting an important bioactive contribution of these metabolites. Interestingly, apigenin-7-*O*-glucoside was previously found to present antibacterial and anti-biofilm effects, with minimum biofilm inhibitory concentration of 0.20 mg/mL against *S. aureus* [69], while the antimicrobial effects of caffeic acid against the same bacterial strain are widely studied [70,71].

### 3.10. Multivariate and Correlations Analyses

To integrate the large dataset obtained for *S. transsylvanica* extracts, pairwise correlation analysis was paired with unsupervised multivariate methods.

**Figure 10:**
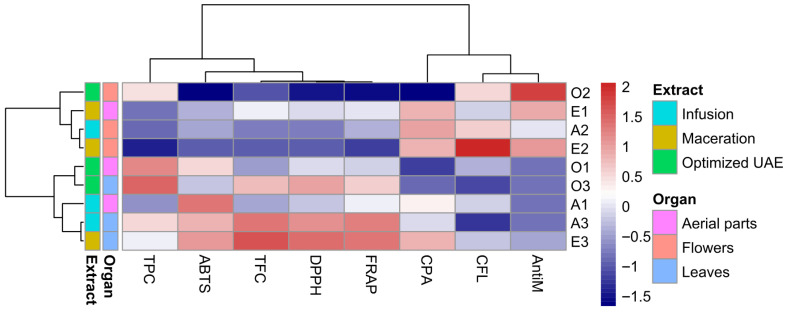
Hierarchical cluster analysis heatmap with dendrogram considering some of the determined phytochemical and bioactive parameters of *S. transsylvanica* extracts. Rows indicate grouping based on extraction type (infusion, optimal UAE, tincture), as well as plant organ (aerial parts, flowers, leaves), with Ward’s minimum variance method and correlation-based distance. Abbreviations: AntiM (antimicrobial potential against *S. aureus*, Kirby-Bauer disk diffusion test), CFL (cumulative flavonoids, HPLC), CPA (cumulative phenolic acids, HPLC), TFC (total flavones and flavonols, AlCl_3_), TPC (total phenolic content, Folin-Ciocâlteu), ABTS/DPPH/FRAP (antioxidant assays).

Hierarchical clustering of standardized variables and correlation analysis (Figure 10) indicated a very strong positive association among antioxidant assays (ABTS, DPPH, FRAP; *r* values between 0.70–0.97), as well as between these assays and both TPC and TFC (for example, DPPH–TFC Pearson correlation was *r* = 0.96). Higher values were generally clustered together for leaf extracts, regardless of the extraction type. Correlations between antimicrobial potential against *S. aureus* (denoted “AntiM”) and chemical variables (cumulative phenolic acids and flavonoids) were generally low, with only modest positive relationships observed for flavonoid-rich extracts. As such, it can be concluded that distinct heatmap clusters were primarily driven by phenolic content and antioxidant capacity. Leaf extracts, regardless of extraction method, formed a compact high-intensity cluster for TFC and all three antioxidant assays, aerial part extracts occupied intermediate positions between leaves and flowers.

**Figure 11:**
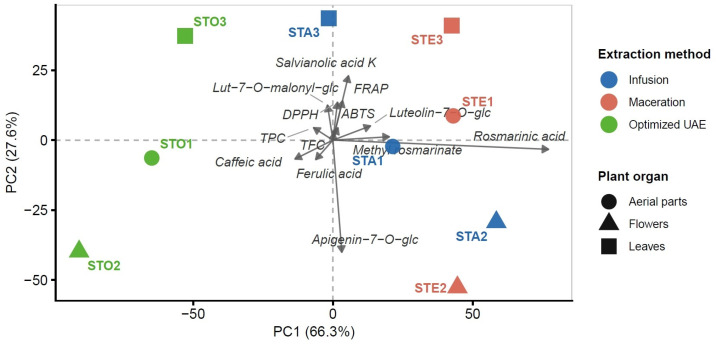
PCA biplot of phytochemical and antioxidant profiles, with two main factors (PC1, PC2) explaining 94% of total variance and Pareto−scaled variables (screening, antioxidant assays, and selected HPLC−MS quantified compounds). Arrows: variable loadings (scaled to scores range). Points: sample scores. Chemical abbreviations: 7-*O*-glc (7-*O*-glucoside), 7-*O*-malonyl-glc (7-*O*-malonyl-glucoside).

The PCA biplot of Pareto-scaled variables (bioactive assays and selected polyphenols abundances, Figure 11) offered a two-dimensional representation of diversity across *S. transsylvanica* extracts, accounting for a high cumulative variance (sum of PC1 and PC2 represented 93.9% of the total, while the sum of PC3 and PC4 only contributed 5.5% to the variance; see also Scree plot in Figure S2—Supplementary Materials). Its interpretation helped to identify dominant chemical patterns across extraction methods and plant organs and to highlight primary differences in polyphenolic abundance.

The first PC, accounting for 66.3% of total variance, separated the samples primarily by extraction efficiency. Several variables (rosmarinic acid, methyl rosmarinate, caffeic acid, ferulic acid, TPC, TFC, and luteolin-7-*O*-glucoside) exhibited loadings projected in the direction of PC1, with the optimized UAE extracts scoring the highest scores (clustered at one extreme of PC1), closely followed by macerates (at the other extreme), and the infusion scoring the lowest overall (except STA2). Thus, these parameters are co-varying based on the extraction protocol, with rosmarinic acid, methyl rosmarinate, and luteolin-7-*O*-glucoside being positively correlated and caffeic/ferulic acids being negatively correlated. Highest loadings in PC1 clearly indicated the preference of hydroethanolic extraction for rosmarinic acid and methyl rosmarinate, while monomer phenolic acids were accumulated during optimized UAE.

The second PC, representing 27.6% of total variance, captured organ-specific differences independently of the extraction method. In this case, flower extracts (i.e., STE2, STO2, STA2) were distantly positioned along PC2 relative to leaf and aerial parts extracts, highlighting the organ-specific source of chemical variation. This was evident in the case of flavonoid glycosides, particularly luteolin-7-*O*-malonyl-glucoside (higher loading for leaves; correlation with ABTS, DPPH, and FRAP) and apigenin-7-*O*-glucoside (highest loading for flowers), with the two variables being negatively correlated along PC2. Moreover, salvianolic acid K showed high loadings toward leaf extracts, with significantly lower abundance in the flower extracts.

The clustering patterns obtained in the PCA biplot reveal two main groups. Firstly, optimized UAE extracts (i.e., STO1, STO2, STO3) consistently clustered toward higher PC1 scores, suggesting a constant and efficient caffeic and ferulic acid recovery across tissues. Secondly, infusions and macerates clustered toward higher rosmarinate derivatives and luteolin-7-*O*-glucoside abundances, while the specific metabolites were apigenin-7-*O*-glucoside (flowers) and salvianolic acid K (leaves).

### 3.11. Study Limitations and Prospects

As expected, phytochemical profiling and extraction optimization efforts present specific challenges and limitations [72,73], which also need to be considered as part of this study. Firstly, harvested material was obtained from a limited geographical location during a single vegetation year, given its infrequent occurrence in the endemic flora; additional environmental factors (such as climatic and seasonal variation, altitude, and soil chemistry) may have also influenced extraction performances [74].

Another limitation stems from the applicability of the optimization to aerial parts, with the subsequent application of optimal conditions across morphologically distinct organs (leaves and flowers). The choice of process variables (ABTS, DPPH, and TPC) presented a methodological limitation, given that classical maceration favored oligomer phenolic acids (rosmarinic, salvianolic acid K), while optimized UAE favored monomer phenolic acids (caffeic, ferulic acid). While TPC was selected as the primary dependent variable for optimization based on several principles (established validity for screening, chemical complexity of the phenolic profile, high-throughput applicability, and TPC–antioxidant correlations) single-marker phenolic quantification would explain the experimental model in more depth. These limitations support further assessment of *S. transsylvanica* by the application of improved methodology, using multiple location harvesting [75], assessment of volatile and non-volatile phytochemical variability [76], optimization using major metabolites (e.g., rosmarinic acid content), and in vivo pharmacological and toxicological determinations.

On the other hand, this study provides the first systematic UAE optimization for *S. transsylvanica*, highlighting a phenolic and antioxidant profile comparable to or exceeding that of widely used medicinal sages from Romanian flora. While infusion and hydroethanolic maceration remain methodologically simpler, UAE offers a much shorter extraction time, reduced solvent use, and selectivity for phenolic acid recovery, supporting its further valorization as a green and efficient alternative [49].

## 4. Conclusions

This study provides the first systematic optimization of hydroethanolic ultrasound-assisted extraction (UAE) for *Salvia transsylvanica*. A D-optimal quadratic design identified 24% amplitude (~5 W), 12 min, and 38% ethanol as optimal UAE conditions for maximizing total phenolic content and antioxidant capacity in aerial parts, with robust model fit and predictive power. When applied to aerial parts, flowers, and leaves, the optimized UAE protocol afforded phenolic-rich extracts with antioxidant activities that were generally comparable to classical aqueous infusions and 70% hydroethanolic macerates, while substantially reducing extraction time and solvent use.

Targeted HPLC-MS analyses confirmed a phenolic profile dominated by rosmarinic acid, salvianolic acid derivatives, caffeic acid, and flavone glycosides, with leaves consistently yielding the highest phenolic levels and antioxidant capacities. Particularly, 70% hydroethanolic maceration was more effective for phytosterols and tocopherols, underscoring a complementary distribution of polar and lipophilic metabolites between modern and traditional extraction approaches. Antimicrobial assays revealed only moderate effects against *Staphylococcus aureus*. Overall, these findings support UAE as an efficient and green strategy for selectively concentrating phenolic antioxidants from *S. transsylvanica*, while highlighting the value of integrating UAE with conventional maceration to capture the broader metabolite spectrum and to further explore the ethnopharmacological potential of this underutilized endemic species.

## Figures and Tables

**Table 1:** The experimental matrix of experimental design employed for UAE optimization and results of experimental runs (data are presented as average of triplicate determinations for dried aerial parts of *S. transsylvanica*). TPC, ABTS, and DPPH were considered as dependent parameters, while ultrasound amplitude, time, and ethanol concentration were used as independent extraction variables.

No.	US Amp. (%) ^1^ X_1_	EtOH (%) X_2_	Time (min) X_3_	TPC (mg GAE/g) Y_1_	ABTS (mg TE/g) Y_2_	DPPH (mg TE/g) Y_3_
1.	20 (−)	20 (−)	5 (−)	40.84	71.95	102.10
2.	60 (+)	20 (−)	5 (−)	44.36	67.51	85.87
3.	20 (−)	20 (−)	15 (+)	39.73	74.16	114.74
4.	60 (+)	20 (−)	15 (+)	41.21	66.52	93.53
5.	60 (+)	80 (+)	5 (−)	15.00	37.39	50.90
6.	20 (−)	80 (+)	15 (+)	20.53	41.19	57.75
7.	60 (+)	80 (+)	15 (+)	18.73	38.94	60.92
8.	20 (−)	50 (0)	5 (−)	43.80	84.50	108.49
9.	20 (−)	80 (+)	10 (0)	19.26	46.34	44.90
10.	40 (0)	80 (+)	5 (−)	18.06	40.30	50.94
11.	60 (+)	50 (0)	10 (0)	43.24	**87.24**	113.20
12.	40 (0)	50 (0)	15 (+)	**45.78**	85.68	107.35
13.	40 (0)	20 (−)	10 (0)	45.62	72.62	101.24
14.	40 (0)	50 (0)	10 (0)	43.82	83.05	**127.92**
15.	40 (0)	50 (0)	10 (0)	44.49	76.40	114.52
16.	40 (0)	50 (0)	10 (0)	40.69	77.50	119.60

^1^ Ultrasound amplitude corresponds to an ultrasonic power of ~4.3 W (X_1_ = 20%), ~9.5 W (X_1_ = 40%), and ~15.6 W (X_1_ = 60%), calculated as average of all experimental runs. Values in bold indicate the highest experimentally observed average for each dependent variable. The coded factor levels were given in parentheses for each parameter, as follows: low-level (−), central (0), and high-level (+).

**Table 2:** Predicted and experimental values of the extract obtained from *S. transsylvanica* aerial parts used for model validation (prior to lyophilization), along with relative recoveries.

Response	Experimental Value	Precision (CV) ^1^	Predicted Value	Relative Recovery ^2^
TPC (mg GAE/g)	50.52 ± 2.38	4.71%	45.51	111.01 ± 3.70%
ABTS (mg TE/g)	90.97 ± 1.29	1.41%	87.10	104.44 ± 1.45%
DPPH (mg TE/g)	149.18 ± 3.48	2.34%	125.55	118.87 ± 3.77%

^1^ Expressed as CV = 100 × (Standard Deviation/Mean); ^2^ Expressed as relative to theoretically predicted values: Relative recovery = 100 × (Experimental/Predicted). Analytical replicate values were expressed as average ± standard deviation (*n* = 3).

**Table 3:** Identification and quantification of phenolic acids and their derivatives in *S. transsylvanica* extracts, expressed as μg metabolite/g of freeze-dried powder.

No.	Compound	Concentration in Each Sample (μg/g dw) ^1^								
		STA1	STA2	STA3	STE1	STE2	STE3	STO1	STO2	STO3
1.	1-*O*-Caffeoyl-beta-D-glucose	13.577	14.698	14.254	13.964	13.433	18.565	9.996	10.074	14.304
2.	3,4-Dihydroxyphenyllactic acid	131.701	131.813	130.441	108.709	91.338	152.050	32.145	17.789	50.763
3.	3,4-Dihydroxyphenyllactic acid-*O*-glucoside	21.621	25.893	9.075	27.057	30.869	11.068	22.703	26.585	10.141
4.	3-Hydroxybenzoic acid	4.511	5.079	4.266	5.788	4.877	5.962	5.289	5.369	5.845
5.	3-*O-*/4-*O*-Methyl gallic acid	0.900	1.074	0.695	ND	ND	ND	1.602	2.054	0.906
6.	Caffeic acid	169.405	170.159	161.285	360.419	363.467	284.486	461.728	631.918	389.733
7.	Caffeoyl-malic acid	13.627	10.513	19.762	12.658	8.500	22.649	ND	ND	10.416
8.	*cis*-Ferulic acid	3.100	3.500	3.100	4.300	4.900	5.000	5.079	6.762	4.877
9.	Coniferyl aldehyde	1.647	1.527	0.406	1.210	0.903	0.616	1.125	0.786	0.363
10.	Ferulic acid	65.320	77.186	53.105	154.478	191.956	102.191	162.833	250.385	130.796
11.	Gallic acid	0.742	2.327	ND	0.581	0.585	0.372	0.068	0.090	0.055
12.	Methyl rosmarinate ^2^	677.904	825.857	637.304	764.414	796.414	860.529	383.390	363.235	487.382
13.	*p*-Coumaric acid	19.492	22.800	21.187	40.907	42.084	40.176	27.087	33.794	31.884
14.	Protocatechuic acid	34.471	49.015	17.162	44.577	61.792	26.190	22.661	29.333	17.239
15.	Protocatechuic acid-4-*O*-glucoside	36.850	30.091	41.398	46.245	34.841	52.218	37.097	34.288	49.815
16.	Rosmarinic acid	17,203.334	19,157.293	15,969.454	18,340.477	18,420.000	17,937.073	13,110.343	11,864.021	13,544.513
17.	Salicylic acid	23.699	25.063	16.427	36.592	37.484	25.794	24.116	25.825	18.673
18.	Salvianolic acid K	497.593	272.023	802.377	455.849	189.384	887.752	282.732	131.909	635.414
19.	Sinapaldehyde	1.268	1.197	0.091	0.700	0.300	0.200	0.300	0.200	ND
20.	Syringaldehyde	1.604	1.901	ND	1.680	1.634	0.600	0.936	0.626	0.431
21.	Syringic acid	8.490	14.918	2.722	10.360	16.508	3.126	4.726	8.957	1.871
22.	Vanillic acid	6.403	9.256	2.947	9.341	10.816	5.857	6.512	9.787	3.895
23.	Vanillic acid-4-O-glucoside	10.854	9.598	11.783	14.228	10.671	15.156	11.855	10.705	13.893
**Cumulative phenolic acids (CPA)**		18,948.111	20,862.780	17,919.241	20,454.532	20,332.753	20,457.629	14,614.321	13,464.492	15,423.208

^1^ ST—*Salvia transsylvanica*. Type of extract: A—aqueous (infusion, 30 min); E—hydroethanolic (70% EtOH, 10-day maceration); O—optimized UAE (24% amplitude, 38% EtOH, 12 min). Plant material: 1—aerial parts; 2—flowers; 3—leaves; ^2^ Rosmarinic acid methyl ester. ND—not detected or below the quantification limit.

**Table 4:** Identification and quantification of flavonoids, flavonoid-glycosides (1–13), and two furanocoumarins (14, 15) in *S. transsylvanica* extracts, expressed as μg metabolite/g of freeze-dried powder.

No.	Compound	Concentration in Each Sample (μg/g dw) ^1^								
		STA1	STA2	STA3	STE1	STE2	STE3	STO1	STO2	STO3
1.	Apigenin	7.763	12.658	0.697	54.923	122.665	5.221	14.360	29.196	1.319
2.	Apigenin 7-*O*-glucoside	1025.400	1575.622	74.388	447.436	2130.265	224.162	940.078	1613.698	77.204
3.	Apigenin-7-*O*-(3″-acetyl)-glucoside	3.100	7.200	ND	4.372	10.151	ND	ND	ND	ND
4.	Apigenin-7-*O*-(6″-acetyl)-glucoside	13.460	28.053	3.975	13.670	25.205	5.027	11.176	24.176	4.418
5.	Eupatorin	7.974	20.744	ND	24.404	56.869	ND	11.053	27.965	ND
6.	Genkwanin	1.755	3.822	ND	11.519	29.492	0.278	3.429	7.767	0.200
7.	Hispidulin	0.392	0.510	0.449	7.074	4.390	6.980	0.926	1.154	1.063
8.	Hyperoside	9.079	8.005	4.163	14.271	12.190	10.029	4.316	2.297	4.074
9.	Isoquercitrin	58.411	57.493	33.100	94.398	95.877	92.450	46.271	40.331	36.578
10.	Luteolin	36.093	59.603	6.063	154.711	237.337	116.325	45.030	79.966	18.273
11.	Luteolin-7-*O*-(6″-*O*-malonyl)-glucoside	282.751	261.091	413.192	240.999	198.604	369.422	262.497	221.442	454.393
12.	Luteolin-7-*O*-glucoside	577.535	570.259	573.916	933.508	841.791	1101.898	503.964	511.915	593.246
13.	Quercetin-3-*O*-neohesperidoside	3.542	4.618	2.561	4.485	5.182	4.056	3.285	3.698	3.051
14.	Esculin	3.955	2.986	5.555	4.782	3.309	6.752	3.226	2.628	5.517
15.	Esculetin	6.954	5.745	7.253	15.528	14.385	13.104	12.515	16.492	12.636
Cumulative flavonoids 1–13 (CFL)		2027.255	2609.678	1112.505	2005.769	3770.019	1935.848	1846.387	2563.604	1193.821

^1^ ST—*Salvia transsylvanica*. Type of extract: A—aqueous (infusion, 30 min); E—hydroethanolic (70% EtOH, 10-day maceration); O—optimized UAE (24% amplitude, 38% EtOH, 12 min). Plant material: 1—aerial parts; 2—flowers; 3—leaves. ND—not detected or below the quantification limit.

**Table 5:** Identification and quantification of some lipidic metabolites in *S. transsylvanica* extracts.

Extract ^1^	Concentration (μg/g dw)				Concentration (ng/g dw)	
	Brassicasterol	Stigmasterol	Beta-Sitosterol	Campesterol	γ-Tocopherol	δ-Tocopherol
STE1	459.3 ± 22.9 ^b^	409.8 ± 4.1 ^b^	5658.6 ± 452.6 ^b^	120.2 ± 4.8 ^b^	1302.2 ± 117.1 ^b^	822.2 ± 73.9 ^a^
STE2	936.4 ± 56.1 ^a^	348.4 ± 17.4 ^c^	6986.5 ± 628.7 ^a^	147.3 ± 7.3 ^a^	361.5 ± 21.6 ^c^	873.5 ± 17.4 ^a^
STE3	18.7 ± 0.3 ^d^	475.1 ± 28.5 ^a^	5397.2 ± 161.9 ^c^	65.8 ± 3.2 ^c^	3690.5 ± 221.4 ^a^	705.1 ± 42.3 ^b^
STO1	ND	ND	135.6 ± 5.4 ^f^	4.4 ± 0.1 ^e^	ND	ND
STO2	18.9 ± 0.9 ^c^	ND	2611.0 ± 18.2 ^d^	7.3 ± 0.2 ^d^	ND	ND
STO3	ND	ND	2053.0 ± 2.1 ^e^	ND	ND	ND

^1^ ST—*Salvia transsylvanica*. Type of extract: A—aqueous (infusion, 30 min); E—hydroethanolic (70% EtOH, 10-day maceration); O—optimized UAE (24% amplitude, 38% EtOH, 12 min). Plant material: 1—aerial parts; 2—flowers; 3—leaves. ND—not detected. Determinations were performed in triplicate and data are presented as mean value ± SD (standard deviation). Different superscript letters correspond to significant statistical differences between values for the same compound (same column), assessed through one-way ANOVA.

## Data Availability

Data is available within the article.

## Supplementary Materials

The following supporting information can be downloaded at: https://www.mdpi.com/article/10.3390/antiox15050561/s1, Table S1. LC-MS/MS protocol details: MS analysis type, precursor ions m/z values, HPLC retention times (min), and calibration curves parameters for analytical standards; Table S2. Phytochemical screening using TPC and TFC (based on AlCl_3_) and in vitro antioxidant potential determination using three assays (ABTS, DPPH, and FRAP); Figure S1. Kirby-Bauer disk diffusion method applied for the determination of antibacterial potential of ST extracts; Figure S2. Scree plot indicating the variance contribution (%) of principal components in PCA.

## Abbreviations

The following abbreviations are used in this manuscript:

ABTS	2,2′-azino-bis(3-ethylbenzothiazoline-6-sulfonic acid) radical scavenging assay
ANOVA	Analysis of variance
APCI	Atmospheric pressure chemical ionization
ATCC	American Type Culture Collection
CFL	Cumulative flavonoids
DoE	Design of experiments
DPPH	2,2-diphenyl-1-picrylhydrazyl radical scavenging assay
dw	Dry weight (after lyophilization)
ESI	Electrospray ionization
FRAP	Ferric reducing antioxidant power
GAE	Gallic acid equivalents
HPLC-MS	High-performance liquid chromatography–mass spectrometry
LC-MS	Liquid chromatography–mass spectrometry
LOD	Limit of detection
LOQ	Limit of quantification
MIC	Minimum inhibitory concentration
MRM	Multiple reaction monitoring
*m*/*v*	Mass to volume ratio
ND	Not detected
PCA	Principal component analysis
QE	Quercetin equivalents
QTOF	Quadrupole time-of-flight (mass spectrometry)
RSM	Response surface methodology
RSP	Response surface plot
SD	Standard deviation
SIM	Selected ion monitoring
STA	Aqueous extract (infusion) of *S. transsylvanica*
STE	Hydroethanolic extract (macerate) of *S. transsylvanica*
STO	Optimized ultrasound-assisted extract of *S. transsylvanica*
TE	Trolox equivalents
TFC	Total flavonoid content
TPC	Total phenolic content
UAE	Ultrasound-assisted extraction
UV	Ultraviolet
*v*/*v*	Volume to volume ratio
