# Supplementary material for: Optimized Ultrasound-Assisted Extraction Reveals Salvia transsylvanica as a Rosmarinic Acid-Rich Endemic Sage with Antioxidant Potential
Source: Antioxidants (Basel). 2026 Apr 28;15(5):561. doi: 10.3390/antiox15050561 (PMC13203183; doi:10.3390/antiox15050561)
Supplement: Supplementary file 1 [file antioxidants-15-00561-s001.zip › antioxidants-4238645-supplementary.pdf]

Supplementary Material – Ultrasound-Assisted Extraction Reveals *Salvia transsylvanica* as a Rosmarinic Acid-Rich Endemic Sage with Antioxidant Potential

Table S1. LC-MS/MS protocol details: MS analysis type, precursor ions *m/z* values, HPLC retention times (min), and calibration curves parameters for analytical standards.

No.	Compound name	Retention time (min)	MS analysis type	Precursor ion, *m/z*	Calibration curve		
					Slope	Intercept	Correlation coefficient
1	Gallic acid	1.36	MRM	169.013	347605	207095	0.9945
2	Protocatechuic acid	2.67	MRM	153.018	338843	9372	0.9999
3	Gentisic acid	3.30	MRM	153.018	290500	71271	0.9987
4	Gallocatechin	2.84	MRM	305.066	167646	77874	0.9964
5	Salicin	4.42	SIM	285.097	60610	-8392	0.9983
6	Procyanidin B3	4.75	MRM	577.134	119245	21579	0.9981
7	Procyanidin B1	5.20	MRM	577.134	103592	-13135	0.9978
8	Procyanidin B4	6.15	MRM	577.134	127379	-28968	0.9928
9	Procyanidin B2	7.40	MRM	577.134	103152	2282	0.9978
10	3-Hydroxybenzoic acid	5.72	MRM	137.023	193277	102262	0.9991
11	Esculin	5.76	MRM	339.071	1423842	141568	0.9990
12	Catechin	5.81	SIM	289.071	266920	69348	0.9988
13	Vanillic acid	6.39	MRM	167.034	201485	67225	0.9965
14	Chlorogenic acid	6.45	MRM	353.087	299848	147143	0.9949
15	Caffeic acid	6.62	MRM	179.034	980373	-359427	0.9924
16	cis-Caffeic acid	6.75	MRM	179.034	788541	254979	0.9984
17	Esculetin	6.68	SIM	177.018	1435952	-253651	0.9990
18	4-O-Caffeoylquinic acid	7.35	MRM	353.087	171751	23491	0.9992
19	Syringic acid	8.08	MRM	197.045	212263	-69487	0.9946
20	Epigallocatechin gallate	8.31	MRM	457.077	224999	-83234	0.9992
21	Epicatechin	8.89	SIM	289.071	334620	152494	0.9995
22	p-Coumaric acid	9.20	MRM	163.039	751021	-259509	0.9982

No.	Compound name	Retention time (min)	MS analysis type	Precursor ion, m/z	Calibration curve		
					Slope	Intercept	Correlation coefficient
23	cis-p-Coumaric acid	9.50	MRM	163.039	809024	-332210	0.9907
24	Procyanidin C1	9.35	SIM	865.198	92509	33249	0.9868
25	Genistin	9.40	MRM	431.097	429750	-239003	0.9934
26	Umbelliferone	9.73	SIM	161.023	2230538	-103378	0.9920
27	Procyanidin A1	9.77	SIM	575.119	176974	46853	0.9958
28	Syringaldehyde	9.85	MRM	181.050	302645	181236	0.9964
29	Salicylic acid	9.99	MRM	137.023	1025057	441422	0.9967
30	Scopoletin	10.95	SIM	191.034	979012	-221054	0.9987
31	Ferulic acid	11.05	MRM	193.050	280746	123106	0.9877
32	cis-Ferulic acid	11.55	MRM	193.050	268216	72126	0.9981
33	Procyanidin A2	12.35	SIM	575.119	140502	-6686	0.9984
34	Coniferyl aldehyde	12.60	MRM	177.055	1403524	766356	0.9977
35	*o*-Coumaric acid	13.47	MRM	163.039	440175	115677	0.9983
36	Sinapaldehyde	13.50	MRM	207.065	670060	93892	0.9972
37	Vitexin	14.25	MRM	431.097	339588	94974	0.9985
38	Vitexin-2-O-rhamnoside	14.78	MRM	577.155	478601	164916	0.9964
39	Hyperoside	15.19	MRM	463.087	397114	17738	0.9986
40	Isoquercitrin	15.55	MRM	463.087	370610	-51847	0.9951
41	trans-Resveratrol	15.47	MRM	227.070	115287	94503	0.9844
42	Rutin	15.81	MRM	609.145	411197	-193332	0.9966
43	Ellagic acid	15.85	SIM	300.998	125556	31465	0.9980
44	Rosmarinic acid	16.45	MRM	359.076	116940	-19157	0.9993
45	Myricetin	16.55	SIM	317.029	358626	-73787	0.9946
46	Hesperidin	16.95	MRM	609.182	321673	130589	0.9981
47	Apigenin 7-glucoside	17.31	MRM	431.097	986652	-282301	0.9948
48	Quercetin-3-*O*-glucose-6-acetate	17.56	MRM	505.098	463225	-130161	0.9992
49	Quercitrin	17.58	MRM	447.092	328523	5502	0.9998
50	Fisetin	17.60	SIM	285.039	540392	-162576	0.9998
51	Kaempferol 3-glucoside	17.70	MRM	447.093	491270	58491	0.9979

No.	Compound name	Retention time (min)	MS analysis type	Precursor ion, *m/z*	Calibration curve		
					Slope	Intercept	Correlation coefficient
52	Ononin	17.97	MRM	429.118	2252767	-105992	0.9933
53	*cis*-Resveratrol	18.16	MRM	227.070	119728	-1070	0.9992
54	Daidzein	18.80	SIM	253.050	1848414	576960	0.9986
55	Quercetin	20.22	SIM	301.034	443217	208552	0.9988
56	Glycitein	20.50	SIM	283.060	1635147	889071	0.9976
57	Luteolin	21.52	SIM	285.039	1079269	-816503	0.9946
58	Genistein	21.60	SIM	269.045	1685128	199947	0.9820
59	Jaceosidin	22.03	MRM	329.066	409652	-73215	0.9998
60	Kaempferol	22.99	SIM	285.039	1081889	-664777	0.9975
61	Coumestrol	23.65	SIM	267.029	1214528	-375427	0.9932
62	Apigenin	23.76	SIM	269.045	1222627	-603580	0.9921
63	Hispidulin	24.05	MRM	299.055	879164	-202732	0.9999
64	Formononetin	25.14	SIM	267.065	2124586	-100846	0.9947
65	Eupatorin	26.60	MRM	343.081	276814	-96261	0.9992
66	Eupatilin	27.05	MRM	343.081	297573	-146528	0.9926
67	Pinocembrin	26.46	SIM	255.065	1852563	717713	0.9985
68	Casticin	27.30	MRM	373.092	429671	-180930	0.9929
69	Chrysin	27.95	SIM	253.050	1815169	-940164	0.9976
70	Galangin	28.56	SIM	269.045	1652480	-545659	0.9968
71	Acacetin	28.70	MRM	283.060	2109562	-770618	0.9972
72	Genkwanin	29.15	MRM	283.060	797884	-73670	0.9987

Table S2. Phytochemical screening using TPC and TFC (based on $AlCl_3$) and *in vitro* antioxidant potential determination using three assays (ABTS, DPPH, and FRAP).

Sample [1]	Yield (% dw)	Phytochemical screening		Antioxidant potential (mg TE/g dw)		
		TPC (mg GAE/g dw)	TFC (mg QE/g dw)	ABTS	DPPH	FRAP
**STA1**	25.43	169.55 ± 6.40^{de}	26.21 ± 1.09^{cd}	231.02 ± 0.42^{a}	533.96 ± 42.01^{bc}	621.08 ± 3.40^{bc}
**STA2**	24.16	161.29 ± 1.78^{e}	25.13 ± 0.61^{d}	193.97 ± 0.47^{d}	487.00 ± 26.39^{bcd}	579.13 ± 5.62^{c}
**STA3**	28.94	206.61 ± 0.80^{bc}	32.69 ± 1.28^{ab}	219.64 ± 0.71^{bc}	656.05 ± 11.31^{a}	741.97 ± 13.88^{a}
**STE1**	17.28	162.80 ± 17.67^{e}	28.19 ± 2.60^{bcd}	195.57 ± 4.85^{d}	545.39 ± 22.94^{b}	618.16 ± 41.50^{bc}
**STE2**	19.37	143.00 ± 4.48^{e}	24.50 ± 1.58^{d}	184.1 ± 6.89^{e}	467.19 ± 24.38^{cd}	495.98 ± 34.72^{d}
**STE3**	18.55	193.16 ± 10.03^{cd}	33.68 ± 1.76^{a}	225.06 ± 4.57^{ab}	679.66 ± 37.35^{a}	752.87 ± 43.82^{a}
**STO1**	11.71	230.62 ± 9.11^{ab}	26.11 ± 1.21^{cd}	213.49 ± 1.75^{c}	543.8 ± 17.79^{b}	602.99 ± 22.48^{bc}
**STO2**	11.56	205.87 ± 4.65^{bc}	24.05 ± 0.05^{d}	169.31 ± 2.61^{f}	420.97 ± 22.22^{d}	457.37 ± 36.85^{d}
**STO3**	11.12	240.06 ± 18.48^{a}	30.46 ± 2.55^{abc}	198.52 ± 2.04^{d}	642.54 ± 3.39^{a}	675.92 ± 4.12^{ab}

[1] ST – *Salvia transsylvanica*. Type of extract: A – aqueous (infusion, 30 min); E – hydroethanolic (70% EtOH, 10-day maceration); O – optimized UAE (24% amplitude, 38% EtOH, 12 min). Plant material: 1 – aerial parts; 2– flowers; 3 – leaves. Results were expressed as average of triplicate ± standard deviation (n = 3) for freeze-dried extracts. Different superscript letters correspond to significant statistical differences between values for the same determination (same column), assessed through one-way ANOVA with post-hoc Tukey's test ($p < 0.05$).

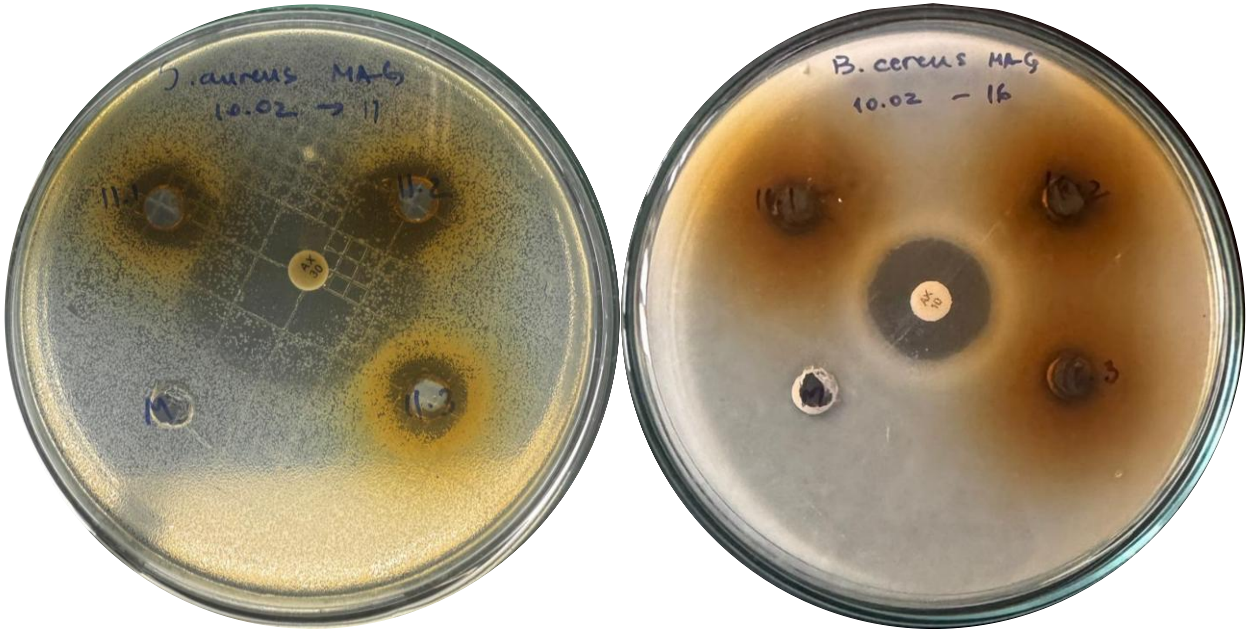

Figure S1. Kirby-Bauer disk diffusion method applied for the determination of antibacterial potential of ST extracts, against *Staphylococcus aureus* (left panel, for SO2; Amoxicillin AX30 control, DMSO 10% negative control) and *Bacillus cereus* (right panel, for SE1; Amikacin AK 10 control, DMSO 10% negative control).

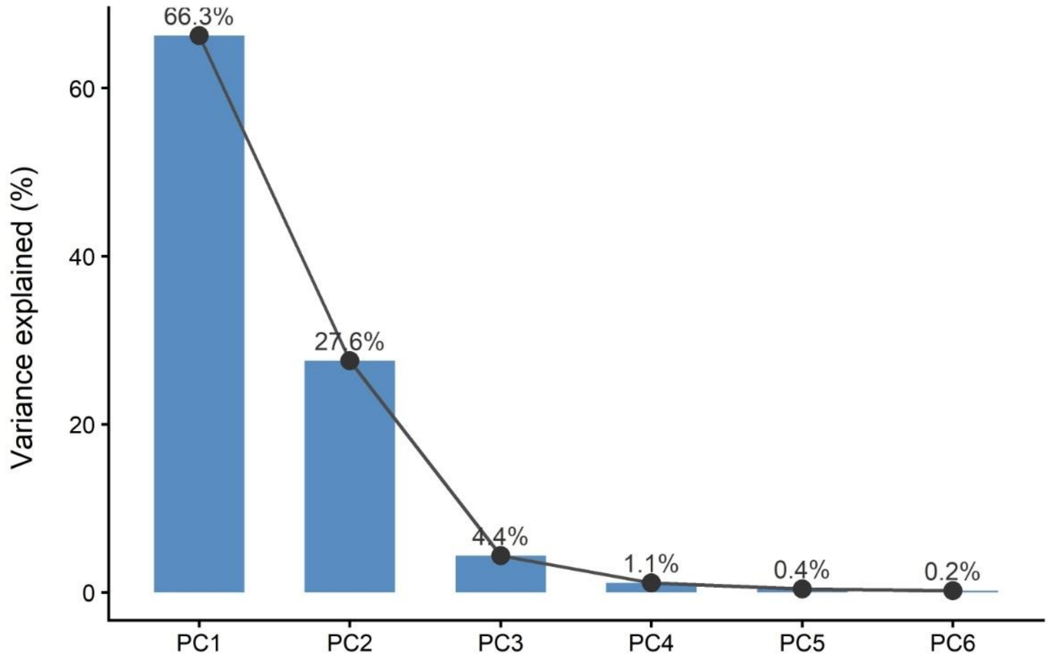

Figure S2. Scree plot indicating the variance contribution (%) of principal components in PCA.
